# Legal Notes for Institutional Workers

**Published:** 1915-01-16

**Authors:** 


### LEGAL NOTES FOR INSTITUTIONAL WORKERS.

The following points of medical interest have arisen in recent Workmen's Compensation cases, and we note them briefly. A workman in the employment of a Board of Guardians as a machinery attendant contracted typhoid fever in the course of his employment, the theory being that it was due to his occupation, the nature of which necessitated the handling of sewage from time to time. He died shortly after contracting the disease, and his widow claimed compensation from the Guardians; but the Court held (*Findlay* v. *Tullamore Union*, 1914, W. C. and Insce. Reports, 222) that while the relationship of workman and employer had existed between the parties, yet as it was not possible to indicate a time and place at which there had been an "accident" which caused the injury, the workman was not entitled to an award under the Act.

The question of medical examination arose in *Smith* v. *Davies and Son, Ltd.*, 1914, W.C. and Insce. Reports, 71. A collier had been injured, but as his employers thought he was better they stopped the compensation. On the miner taking action for recovery of it they required him to submit to medical examination. He refused, and the Court held that the proceedings must be suspended until such examination had taken place.

A novel point in this branch of law arose in *Della Rocca* v. *Stanley Jones and Co.*, 1914, W.C. and Insce. Reports, 33. A workman broke his arm while working at his employment. His employers sent him to a hospital, but owing to unskilful surgical treatment there his arm did not and could not completely recover. After doing light work for some time at full wages the man was dismissed, and then took proceedings claiming compensation. The Court found that his employers could not be held responsible for unskilled treatment at the hospital to which they had sent the injured employee, and that as the workman's present incapacity for work resulted from the unskilful treatment at the hospital, and not from the injury, he was not entitled to recovery of compensation.

In *Flynn* v. *Bengers and Others*, 1914, W.C. and Insce. Reports, 238, it appeared that the applicant for compensation had been examined by a doctor on behalf of the employers. The doctor had not been called as a witness, but a certificate given by him was put in and received as evidence and acted on by the County Court Judge. On appeal it was held that this certificate was wrongly admitted as evidence.

With regard to doctors' certificates, there is also the case of *Mapp* v. *Straker and Son*, W.C. and Insce. Reports, 98, in which a certifying surgeon under the Factory and Workshops Act certified, as prescribed by the Workmen's Compensation Act, that a workman was suffering from an industrial disease—namely, lead-poisoning or its *sequelæ*—but the certificate stated the symptoms of the disease to be indefinite, and only to be inferred from the history given by the patient. Such a certificate, it was decided, was too ambiguous and contradictory to justify giving compensation to the workman.

The case of *Lewis* v. *Port of London Authority*, W.C. and Insce. Reports, 299, is the leading case on the question of whether death occurring some time after an accident is due to the accident. The facts were briefly as follows. A workman fifty-one years of age died from the effects of an operation upon a malignant tumour of the kidneys. About sixteen months before his death he met with a severe accident in his employment, a heavy strut falling on his back near the region of the kidneys. After this accident he was away ill for about four months. Then he returned to work for about seven months, but went into hospital, when the tumour was discovered and excised. He ultimately died from the effects of the operation. The medical evidence was that the tumour might have been congenital, or might have been caused by the blow, or might have been made malignant by the blow or by other causes. There was no evidence of any other cause except the accident. There was evidence that prior to the accident the deceased was a strong, healthy man having no trouble with his kidneys, and that after the accident he was never able to do his work as he had done it before the accident. The Court found that the accident caused the tumour which then existed in a quiet condition to become malignant, and that the death resulted from injury by accident arising out of and in the course of the employment, therefore justifying an award of compensation.

